# Strategy for communicating benefit-risk decisions: a comparison of regulatory agencies' publicly available documents

**DOI:** 10.3389/fphar.2014.00269

**Published:** 2014-12-04

**Authors:** James Leong Wai Yeen, Sam Salek, Stuart Walker

**Affiliations:** ^1^Center of Regulatory Excellence, Duke-NUS Graduate Medical SchoolSingapore, Singapore; ^2^Department of Pharmacy, University of HertfordshireHatfield, UK; ^3^Institute for Medicines DevelopmentCardiff; ^4^Centre for Innovation in Regulatory ScienceLondon, UK

**Keywords:** benefit-risk communication, benefit-risk assessment, regulatory agency, documentation, template

## Abstract

The assessment report formats of four major regulatory reference agencies, US Food and Drug Administration, European Medicines Agency, Health Canada, and Australia's Therapeutic Goods Administration were compared to a benefit-risk (BR) documentation template developed by the Centre for Innovation in Regulatory Science and a four-member Consortium on Benefit-Risk Assessment. A case study was also conducted using a US FDA Medical Review, the European Public Assessment Report and Australia's Public Assessment Report for the same product. Compared with the BR Template, existing regulatory report formats are inadequate regarding the listing of benefits and risks, the assigning of relative importance and values, visualization and the utilization of a detailed, systematic, standardized structure. The BR Template is based on the principles of BR assessment common to major regulatory agencies. Given that there are minimal differences among the existing regulatory report formats, it is timely to consider the feasibility of a universal template.

## Introduction

The evolution of the requirements for assessing the benefits and risks of medicinal products has resulted in changes in the regulatory review process. Beyond the separate assessment of benefits and risks, the emphasis is now on the balance between the two, having to justify the potential harms in view of the efficacy claims (Breckenridge, 2010). This balance can be expressed in a transparent manner using a structured framework that aids in the communication of the differences in opinions between regulators and the pharmaceutical industry. In a changing society where the demand is for transparency of such decision-making processes, there is now a major challenge to adequately communicate the relevant information to stakeholders.

As expectations of stakeholders change with the rapid advancement of science, regulatory agencies need to adapt to meet these changing requirements. In the European Medicines Agency (EMA) *Roadmap to 2015*, one of the strategic areas identified was the reinforcement of the benefit-risk balance assessment model through a set of priority activities (European Medicines Agency, 2011a). These included looking at appropriate quantitative tools, improving the quality and consistency of the outcomes, and reviewing the European Public Assessment Reports (EPARs) to improve communication of benefit-risk decisions to stakeholders and to increase the involvement of patients, academia and healthcare professionals. Similarly, since 2009 the US Food and Drug Administration (FDA) have begun initiatives to explore systematic approaches to assess and communicate benefits and risks. Their initiatives have included the development of a framework to characterize and provide a structure for the benefit-risk assessment already existing in their decision-making processes, as well as communicating the reasoning behind the decision to all stakeholders (US Food and Drug Administration, 2012), which has led to the current FDA five-step benefit-risk framework. These steps are related to the five key areas to be discussed in the assessment of a medicine, namely the analysis of the condition, the medical need for the product, clinical benefits, risks and risk management. Mullin, commented that this structured framework has the potential to improve the predictability and consistency of decision-making as it is capable of clearly outlining both the available evidence and the uncertainties (Centre for Innovation in Regulatory Science, 2011). This would enable the US FDA to articulate the consideration and clinical judgment taken for the benefit-risk decision and hence improve the transparency of the decision-making process (US Food and drug Administration, 2013). The US FDA has also published its own user's guide on communicating benefits and risks, which provides the expectations and standards for communication (US Food and drug Administration, 2011). Similarly, Australia's Therapeutic Goods Administration (TGA) will be focusing on increasing transparency through their new initiatives and engaging stakeholders with a new framework for communicating the benefits vs. risks approach in their regulation of medicines (Therapeutic Goods Administration, 2013). This increase in transparency is to be achieved through information that is easily understood by patients and consumers and shared with healthcare professionals. TGA aims to provide accessible, clear and consistent relevant information through various multimedia platforms.

The results from a recent study (Leong et al., 2013) showed that both regulatory agencies and pharmaceutical companies believe that a benefit-risk framework would enhance the quality (transparency and consistency) of communication and should provide documentation for a structured discussion, acting as a tool for communication among peers within an organization and between the organization and other stakeholders (Centre for Innovation in Regulatory Science, 2012). This eight-step universal benefit-risk framework encompasses the principles of existing frameworks by other major regulatory agencies such as the US FDA (US Food and drug Administration, 2013) and EMA (European Medicines Agency, 2010) (Table 1).

**Table 1:** **Comparisons of US FDA and EMA benefit-risk assessment frameworks with the Universal Benefit-Risk Framework**.

**Frameworks reviewed**	**Core elements**							
	**Framing the decision**	**Identifying benefits and risks**		**Assessing benefits and risks**		**Interpretation and outcome**		
US FDA	Analysis of conditions and unmet medical needs	Clinical benefits, risks	Evidence and uncertainties					Conclusions and reasons, risk management plans
EMA PrOACT-URL	Nature and framing of the problem	Objectives, favorable and unfavorable effects		Alternatives regarding options to be evaluated and the consequences	Trade-offs and benefit-risk balance	Evaluating uncertainty	Effects table and risk tolerance	Consistency of decisions (linked decisions)
	**Step 1**	**Step 2**	**Step 3**	**Step 4**	**Step 5**	**Step 6**	**Step 7**	**Step 8**
Universal benefit-risk framework	Decision context	Building the value tree	Customizing the value tree	Weighting (relative importance) of benefits and risks	Scoring the options	Evaluating uncertainties	Concise presentation of results (visualization)	Expert judgment and Communications

In collaboration with CIRS, TGA, the Singapore's Health Sciences Authority (HSA), Health Canada and SwissMedic established a four-member Consortium on Benefit-Risk Assessment (COBRA), which aimed to develop a systematic qualitative approach for the benefit-risk assessment of medicines in order to facilitate joint and shared reviews. A benefit-risk (BR) documentation template, or BR Template, was developed based on the EMA reflection paper (European Medicines Agency, 2008) and reviewed by the Consortium through both retrospective and prospective studies employing its use, with plans for making the template more reflective of actual practice. This BR Template was designed to enhance effective documentation and communication of decisions and was used as the basis of a comparison in this study. The summary portion of this template was extracted to produce a “stand-alone” BR Summary Template, with the objective that this simplified template would suffice to meet the needs of jurisdictions with emerging pharmaceutical markets. This study aims to review the publicly available assessment reports to see if they adequately fulfill the functions found in the BR Template and BR Summary Template.

The objectives of this study were to:

- Compare the format of publicly available assessment reports from US FDA, EMA, Health Canada and TGA with the BR Template and BR Summary Template
- Evaluate whether these four regulatory agencies have an effective approach to communicating benefit-risk decisions to all stakeholders
- Examine the utility of the BR Summary Template for communicating benefit-risk decisions by the US FDA, EMA and TGA using a case study approach

## Methods

### Development of the BR template and BR summary template

A benefit-risk documentation template, or BR Template, was developed based on the EMA reflection paper (guidance document, 2008). This document contained those elements considered essential to the assessment of benefits and risks of medicines. These elements were then transformed into a template that allowed systematic documentation and editing. This initial developmental template was then reviewed against the universal framework so that it could support its underlying principles. The template comprises six sections namely: Section 1, *Background* (decision context); Section 2, *Overall Summaries* (quality, non-clinical, human pharmacology, clinical); Section 3, *Identified Benefits and Risks* (list of all benefits, list of all risks); Section 4, *Benefit-Risk Study Findings* (benefits observed in pivotal and non-pivotal studies, overall summary of risks, adverse effects/events, and uncertainties); Section 5, *Benefit-Risk Summary Table and Expert Judgment* (benefits—weighting/relative importance and valuing, risks—weighting/relative importance and valuing); and Section 6, *Benefit-Risk Conclusions*. This initial template was assessed by the Consortium who evaluated its use in a feasibility study, which led to minor amendments of the template incorporating all the feedback from the Consortium. This BR Template was then applied in a retrospective study followed by a prospective study for the review of a number of products, which resulted in the final version of the Benefit Risk Template.

It was considered beneficial by the Consortium that the addition of a Summary section to the BR Template would enhance its internal and external value for communication purposes to stakeholders. Therefore, a Summary section was developed by the expert reviewers who identified the key features from the BR Template (benefit-risk conclusions, decision context, identified benefits and risks, benefit-risk: weighting/relative importance and valuing and benefit-risk management). It was later recognized that the BR Template was going to be more relevant to the review process employed by the established agencies, but the Summary Template, as a shorter version, would be of more benefit to the maturing agencies. The Summary Template has subsequently been validated by several agencies that have carried out retrospective reviews of a number of products.

### Comparison of the publicly available documents from four reference agencies

In order to establish the utility of the BR Template, reference agencies were selected based on the criteria of a positive history, global recognition and the public availability of assessment reports. For the purpose of comparison in this study, the following report formats of the four reference agencies were selected:

- US FDA—Medical Review and the Benefit-Risk Assessment
- EMA—European Public Assessment Report and the Executive Summary
- Health Canada—Summary Basis of Decision
- TGA—Australian Public Assessment Report

Report formats were retrieved online for each agency and where appropriate, a recent publicly available assessment report would be used to review the contents of the format or support the understanding of the format. A comparison of the report formats from the four reference agencies was conducted by reviewing the section headings of the report against those of the BR Template and BR Summary Template. Where there was a summary in the reference agency format, this would be directly compared with the BR Summary Template and the findings are tabulated and presented.

Furthermore, to illustrate the use of the BR Summary Template, a case study was conducted using a recent US FDA Medical Review (European Medicines Agency, 2013a), EPAR (Therapeutic Goods Administration, 2014) and AusPAR (European Medicines Agency, 2011b) for the same product. Ziv-aflibercept, indicated for the treatment of metastatic colorectal cancer, was chosen as it was approved around the same time by the agencies (03 August 3, 2012 for US FDA, February 1, 2013 for EMA and April 2, 2014 for TGA). Importantly, the US FDA Medical Review was written according to the new five-step benefit-risk framework that features the Benefit-Risk Assessment. These two respective summaries were transferred into the BR Summary Template and the omissions reviewed. This entire study was designed to be exploratory and the outcomes were interpreted to provide qualitative inferences relating to the aims. No statistical analyses were planned or conducted.

## Results

The outcomes are presented in three parts:

- Part I—Formats of the four reference agencies' publicly available reports
- Part II—Comparison of the four reference agencies' report formats with the BR Template and BR Summary Template
- Part III—Case study of US FDA, EMA and TGA summary reports on ziv-aflibercept (Zaltrap®; Regeneron Pharmaceuticals, Inc./Sanofi-Aventis U.S. LLC, Bridgewater, NJ, USA)

### Part I—formats of reference agencies' publicly available reports

#### US FDA medical review

The US FDA Medical Review consists of nine sections (Table 2), with the opening section presenting the recommendations and benefit-risk assessment (based on the five-step benefit-risk framework). The remaining sections present the details of the assessment supporting the recommendations. It is known that the publicly available reports from US FDA are a redacted subset of the complete evaluation data. The original dataset would have included discussions of queries and responses by the sponsor with the US FDA.

**Table 2:** **Format of US FDA medical review**.

**Section**	**Content**		
1	Recommendations/ Risk benefit assessment		
2	Introduction and regulatory background	Availability of proposed active ingredient in US	Summary of pre-submission regulatory activity related to submission Other relevant background information
	Product information	Important safety issues with consideration to related drugs	
	Tables of currently available treatment for proposed indications		
3	Ethics and good clinical practices	Compliance with GCP	
	Submission quality and integrity	Financial disclosures	
4	Significant efficacy/safety issues related to other review disciplines	Clinical microbiology	Clinical pharmacology (mechanism of action, pharmacodynamics, pharmacokinetics)
	Chemistry manufacturing and controls	Preclinical pharmacology/toxicology	
5	Sources of clinical data	Review strategy Discussion of individual studies/clinical trials	
	Tables of studies/clinical trials		
6	Review of efficacy	Protocol violationsAnalysis of primary endpoints	Subpopulations
	Efficacy summary	Analysis of secondary endpoints	Analysis of clinical information relevant to dosing recommendations
	Indication (methods, demographics, subject disposition)	Other endpoints	Additional efficacy issues/analyses
7	Review of safety		
	Safety summary		
	Methods (studies, categorization, pooling of data)		
	Adequacy of safety assessment (over all exposure, dose response, special animal and/or *in vitro* testing, metabolic/clearance/interaction workup, potential AE for similar drugs)		
	Major safety results (deaths, non-fatal SAE, dropouts/discontinuation, significant AE, specific primary safety concern		
	Supportive safety results (common AE, lab findings, vital signs, ECGs, special safety studies, immunogenicity)		
	Other safety explorations (dose dependency, time dependency, drug-demographic/drug-disease/drug-drug interactions		
	Additional safety evaluations (human carcinogenicity, human reproduction/pregnancy data, pediatric and effects on growth, overdose/abuse potential/withdrawal/rebound		
	Additional submissions/safety issues		
8	Post-market experience		

The US FDA five-step benefit-risk framework appears to closely reflect the format of its benefit-risk Assessment (Table 3), and therefore was compared with the BR Summary Template. Although the five-step benefit-risk framework may appear less comprehensive than other existing frameworks, the US FDA is currently reviewing a list of questions that should be included under each of these steps, in an approach similar to the EMA guidance for the assessment of benefits and risks.

**Table 3:** **US FDA Benefit-risk framework**.

**Decision factor**	**Evidence and uncertainties**	**Conclusions and reasons**
Analysis of condition		
Current treatment options		
Benefit		
Risk		
Risk management		
Benefit-risk summary assessment		

#### EMA EPAR

The EPAR consists of an Executive Summary and four sections (Table 4). The publicly available EPAR is extracted from the complete assessment report which would have included responses and justifications to EMA for queries raised.

**Table 4:** **Format of EMA EPAR**.

	**Executive Summary**		
1	**Background information on the procedure**	**Submission of the dossier**	**Steps taken for the assessment of the product**
2	**Scientific discussion and introduction**		
	**Quality aspects**	**Clinical aspects**	**Clinical safety**
	Introduction	Introduction	Discussion on clinical safety
	Active substance	Pharmacokinetics	Conclusion on clinical safety
	Finished medicinal product	Pharmacodynamics	Pharmacovigilance
	Discussion on chemical, pharmaceutical and biological aspects	Discussion on clinical pharmacology	User consultation
	Conclusions on the chemical, pharmaceutical and biological aspects	Conclusion onclinical pharmacology	
	Recommendations for future quality development		
	**Non-clinical aspects**	**Clinical efficacy**	
	Introduction	Dose response studies	
	Pharmacology	Main studies	
	Pharmacokinetics	Supportive studies	
	Toxicology	Discussion on clinical efficacy	
	Ecotoxicity/environmental risk assessment	Conclusion on clinical efficacy	
	Discussion on non-clinical aspects		
	Conclusion on non-clinical aspects		
3	**Benefit-risk balance**		
4	**Recommendations**		

#### Health Canada Summary Basis of Decision

The Health Canada's Summary Basis of Decision (SBD) consists of eight sections (Table 5). This is a publicly available document that presents the relevant information to support the decision made by Health Canada for the product (Health Canada, 2012a). Unlike the US FDA Medical Review and the EPAR, there is no separate summary portion, as the SBD is meant for this purpose.

**Table 5:** **Format of Health Canada summary basis of decision**.

**Section**	**Content**	**Purpose**
PAAT	Post-Authorization	List of post-authorization activities for the approved product
	Activities Table	
1	What was approved?	Information on approved indication, intended population, contraindications and product presentations
2	Why was <product> approved?	Discussion on basis of benefit-risk balance
3	What steps led to the approval of <product>?	Submission milestones
4	What follow-up measures will the company take?	Information on post-approval commitment
5	What post-authorization activity has taken place for <product>?	Information provided as link to earlier section on
		Post-Authorization Activity Table (PAAT)
6	What other information is available about drugs?	Links to other webpages within Health Canada website
7	What was the scientific rationale for Health Canada's decision?	Details on:
		a) Clinical Basis of Decision
		i. Clinical pharmacology
		ii. Clinical efficacy
		iii. Clinical safety
		iv. Safety topics of special interest
		b) Non-clinical Basis of Decision
		c) Quality Basis of Decision

#### TGA Australian Public Assessment Report

The TGA Australian Public Assessment Report (AusPAR) consists of six sections (Therapeutic Goods Administration, 2012) (Table 6), the format being close to the EPAR but without the Executive Summary. As with the formats of the other three agencies, agency-specific information is that related to individual regulatory and submission information. It is known that the AusPAR contains information extracted from the complete, original assessment reports.

**Table 6:** **Format of TGA AusPAR**.

**Section**		**Content**
1	Introduction to product submission	Regulatory status
	Submission details	Product information
	Product background	List of abbreviations
2	Quality findings	Biopharmaceutics
	Drug substance	Advisory committee considerations
	Drug product	Quality summary and conclusions
3	Non-clinical findings	Pharmacokinetics
	Introduction	Toxicology
	Pharmacology	Non-clinical summary and conclusions
4	Clinical findings	Dosage selection for pivotal studies
	Introduction	Efficacy
	Pharmacodynamics	Safety
	Pharmacokinetics	Clinical summary and conclusions
5	Pharmacovigilance findings	Risk management plan
**OVERALL CONCLUSION AND RISK/BENEFIT ASSESSMENT**		
6	Background	Risk management plan
	Quality	Risk-benefit analysis
	Non-clinical	Outcome
	Clinical	

### Part II—comparison of the four reference agencies' report formats with the BR template and BR summary template

The formats of the reference agencies' reports are generally similar when compared with the BR Template (Walker et al., 2014). They were all found to lack the features that list: (1) the identified benefits and risks; (2) application of values and weights (relative importance); and (3) visualization of the assessment outcomes (Supplementary material). In addition, while it is acknowledged that relevant discussions and considerations contributing to the final benefit-risk decision maybe reported in the existing reference agencies' reports, the BR Template allowed for this through a structure of guided questions.

In comparison with the BR Summary Template, it was found that only two reference agencies have defined summaries within the report. The US FDA Medical Review has the Recommendations/Benefit-Risk Assessment, which is a discussion based on the benefit-risk framework employed by US FDA. The Executive Summary of the EMA EPAR does not have a structure and presents the information in a general discussion. The entire Health Canada SBD and TGA AusPAR were compared to the BR Summary Template, as it is the intent of both to function as summaries of the actual assessment reports. Details of the comparison with the BR Summary Template are found in Annex B in (Supplementary Material).

#### US FDA Medical Review

The approach of the US FDA Medical Review and the BR Template was found to be similar, with the focus on the contribution of clinical efficacy and safety to the overall benefit-risk balance and a significant contribution of quality, non-clinical and pharmacology concerns succinctly discussed.

Overall, it was observed that the US FDA Medical Review was designed to present details of the evaluation processes including those of the studies and considerations, while the BR Template presents only the information that will directly contribute to the decision on the benefit-risk balance. This can be seen in the detailed structure of the US FDA Medical Review, compared with the more concise BR Template. In terms of utility, the BR Template and BR Summary Template appear to share the US FDA Medical Review's capability to present critical information regarding the benefit-risk decision. The more explicit display using the BR Template's method of listing identified benefits and risks, use of weighting, valuing and visualization may facilitate an improved outcome through a more structured format on the discussion for benefit-risk balance and therefore enhance communication to stakeholders for whom this information is important. Comparison with the BR Summary Template behaved exactly the same as the BR Template exhibiting the same attributes.

#### EMA EPAR

In assessing the benefit-risk balance, the BR Template provided a more structured format through the use of guiding questions, while the EPAR was a general descriptive write-up. The deficiencies seen in the EPAR were found to be similar to those observed for the US FDA Medical Review.

The Executive Summary of the EPAR was compared with the BR Summary Template, which presents structured, concise information leading to the benefit-risk decision, exceeding the utility of the Executive Summary in the EPAR. Therefore, the BR Summary Template may communicate the outcomes in a more transparent manner than the Executive summary in the EPAR.

#### Health Canada SBD

In the assessment of efficacy and safety, it appears that the SBD does not provide a detailed structure in presenting information compared with the BR Template. Overall, both the SBD and BR Template are comparable for the documentation of clinical efficacy, safety and benefit-risk assessment. The SBD, however, did not provide a list of benefits and risks that were considered and subsequently excluded or included in the benefit-risk assessment. Again, weighting (relative importance), valuing and visualization were not included, which would improve transparency as well as communicating the basis of decision. These deficiencies were similarly observed when the SBD was compared with the BR Summary Template.

#### TGA AusPAR

As observed in the other three publicly available documents, the AusPAR was deficient in that it did not provide a list of benefits and risks that were considered, nor did it include their relative importance, their values, or any visualizations. As observed with the other agencies, additional features of the BR Template may help increase the effectiveness of discussion and communication.

It was observed that there was no defined summary for the AusPAR, the section on overall conclusion and the benefit-risk assessment appears to function similarly to the US FDA Recommendations/Benefit-Risk assessment and EPAR's executive summary. The AusPAR appears to represent most of the functional sections of the BR Summary Template, although it still has the deficiencies detailed above.

### Part III—case study of the US FDA, EMA and TGA summary reports on Ziv-aflibercept

Ziv-aflibercept was approved by both US FDA and EMA and the publicly available Medical Review (US Food and Drug Administration. Medical Review, 2012) and EPAR (European Medicines Agency, 2013a) were retrieved from the internet. Both of these documents provided similar information (Table 7), but presented in a different manner. The Executive Summary was written in a continuous descriptive prose but the US FDA Benefit-Risk Assessment was presented under six headings. Overall, the BR Summary Template is more structured in presenting the information for the benefit-risk decision.

**Table 7:** **Case study using Ziv-aflibercept—comparison of US FDA, EMA and TGA summaries with BR summary template**.

**BR Summary template**	**US FDA**	**EMA**	**TGA**
**Content**	**Risk benefit assessment**	**EPAR—executive summary**	**AusPAR—overall conclusion**
1.1 Background (Decision context)			
1.1.1 Specify proposed therapeutic indication	✓	✓	✓
1.1.2 Treatment modalities evaluated	✓	✓	✓
1.1.3 Medical need	✓	✓	✓
2.1 Overall summaries			
2.1.1 Quality conclusions	Not available	Not available	✓
2.1.2 Non-clinical conclusions	Not available	Not available	✓
2.1.3 Human pharmacology conclusions	Not available	Not available	✓
2.1.4 Clinical conclusions	✓	✓	✓
3.1 Identified benefits and risks			
3.1.1 Listing of all benefits, and justification for inclusion and exclusion	Not available	Not available	Not available
3.1.2 Listing of all risks, and justification for inclusion and exclusion	Not available	Not available	Not available
4.1 Clinical study summary	✓	✓	✓
5.1 Risks: Overall summary	Not available	Not available	✓
6.1 Weighting and valuing of benefits and risks	Not available	Not available	Not available
7.1 Conclusion			
7.1.1 For negative benefit-risk balance, discussion on the harm	Not applicable	Not applicable	Not applicable
7.1.2 Discussion on evolution of the benefit-risk balance	Not available	✓	✓
7.1.3 Discussion on outstanding issues and other significant information (hearings, advisories, patients, consumers, stakeholder inputs)	Not available	Not available	✓
7.1.4 Discussion on pharmacovigilance plans and risk mitigation plans	✓	Not available	✓
7.1.5 Discussion on need for further studies	✓	✓	✓
7.1.6 Any other information relevant to the benefit-risk decision	✓	Not available	✓
7.1.7 Conclusion on the benefit-risk balance for proposed indication	✓	✓	✓
7.1.8 Recommendation indication	✓	✓	✓

#### US FDA benefit-risk assessment

The BR Summary Template was completed with the information (Annex C in Supplementary Material) from the Benefit-Risk Assessment for ziv-aflibercept. The decision context of the BR Summary Template could be sufficiently completed with information from this report. Conclusions on quality, non-clinical, and human pharmacology were absent from the Benefit-Risk Assessment.

The benefits were included for the benefit-risk assessment but no reasons were provided for their inclusion. There was no information provided on those benefits that were reviewed but subsequently excluded. Safety parameters included could be inferred by the reasons provided in the Benefit-Risk Assessment but the risks reviewed and subsequently excluded were not documented. Weighting (relative importance) and valuing were not documented and there were no specific comments on the uncertainties relating to the listed benefits and risks. The section of the BR Summary Template (Section 7 of Annex B in Supplementary Material) could be completed from this report although not presented in a structured manner. Overall, application of the BR Summary Template to the US FDA Benefit-Risk Assessment provided a more structured and guided discussion of the decisions leading to the eventual benefit-risk balance.

#### EPAR executive summary

The BR Summary Template was completed with the information (Annex C in Supplementary Material) from the EPAR Executive Summary. The Executive Summary has no structure and is presented in a single section. The quality, non-clinical, human pharmacology conclusions of the BR Summary Template could not be completed as they were absent from the Executive Summary and this is a similar situation to that observed with the US FDA Benefit-Risk Assessment. As there was no specific safety summary, the clinical conclusion of the BR Summary Template remained incomplete. The benefits included for benefit-risk assessment are presented in the Executive Summary of the EPAR but there were no reasons for their inclusion. In addition, there were no indications for those benefits excluded or reasons for their exclusion. A similar pattern was observed for the safety parameters.

Sufficient information was provided in the Executive Summary to complete the BR Summary Template clinical study information and tables, but no weights, values or comments on uncertainties were available. The above observations are expected as relative importance is carried out implicitly but not explicitly in many agencies. However, the safety exposure information was written entirely as a paragraph and could not be uploaded as an image into the BR Summary Template. The structured discussion of the BR Summary Template was not adequately completed using the Executive Summary.

#### TGA overall conclusion and risk/benefit assessment

The BR Summary Template was completed with information from the Overall Conclusion (Annex C in Supplementary Material). This last section of the main AusPAR (Therapeutic Goods Administration, 2014) is organized into seven headings, namely background, quality, non-clinical, clinical, risk management plan, benefit-risk analysis and outcome. Compared with BR Summary Template, it appears that it may have an advantage over this summary by expressing the required information in a succinct way.

## Discussion

The comparisons conducted in this study have shown that the formats of the publicly available assessment reports from the four reference agencies are similar and generally allow the information generated through the course of the evaluation to be described. The differences between these reports are largely related to their format arrangement and headings provided for each section. While there is no universal template for an assessment report, there does not appear to be major differences as to how such information should be presented. Thus, given the commonalities with their presentation, only minor changes are required to their current formats in order to achieve a potential universal standard structure.

The publicly available assessment reports are the means for documenting the relevant information made available to stakeholders and to communicate the basis and justification for BR decisions. The US FDA has made a recent attempt through PDUFA V, to provide a Benefit-Risk Assessment, based on their five-step framework, which details their considerations contributing to the regulatory decision and features an additional succinct benefit-risk assessment summary. EMA has commissioned an external expert to improve its communication of benefits and risks (European Medicines Agency, 2011b) and the EPAR features an Executive summary to provide concise information. Health Canada has completed its two phases of the initiative to improve documentation and communication to the public, with an emphasis on the discussion of the benefit-risk balance and the basis of the decision (Health Canada, 2012b). Likewise, TGA has commenced a project targeted at improving communication of information to patients and physicians. Therefore, it is concluded that these agencies recognize the need to effectively communicate the basis of their decisions through a concise documentation tool.

The primary concern of most patients would be to know if a product is effective and safe, while the physicians would want to know the details to make a better informed decision when choosing an optimal treatment for their patients. Therefore, clarity in the presentation of such information is of paramount importance. For pharmaceutical companies, a documented transparent decision-making process would enable them to understand the basis of the regulatory decision, the rationale for the inclusion or exclusion of benefits and risks, as well as the final benefit-risk balance. This would, therefore, provide a suitable platform to discuss any discrepancies in interpretation or differences in opinions. In evaluating the product for pricing and reimbursement, HTA agencies would also want to understand the rationale for the approval of a product. A failure to achieve this understanding might affect a product's accessibility for patients and influence the healthcare delivery in terms of cost and clinical management. Assessment reports of major regulatory agencies are often accessed by smaller agencies in the emerging markets to support their local decisions and thus these regulatory agencies should also be considered as key stakeholders for the publicly available assessment reports.

Certain jurisdictions may require publication of the assessment reports as a move to increase the transparency of the decision-making processes while others may require varying amounts of information to be made public. As previously discussed, it is also not known if the current practices of providing the publicly available assessment reports actually achieve the transparency required or desired by the stakeholders as there are no studies describing this type of feedback from pharmaceutical companies, physicians, patients or regulatory agencies. In fact, the vast amount of unstructured information provided may possibly hamper understanding and thus communication. The use of summaries like the Executive Summary of the EPAR, the Benefit-Risk Assessment of US FDA and Overall Conclusion of the AusPAR aims to further improve communication. However, as seen in this case study of ziv-aflibercept, a more structured and guided discussion, such as that provided by the BR Summary Template, may further help to improve both transparency and communication and prevent the omission of information assessed by the reviewer and deemed important to stakeholders. The comparison of the summaries showed that there are elements missing which could facilitate effective communication. As such, the elements from the BR Summary Template found missing in the summaries of the reference agencies may serve as a starting platform to enhance the effectiveness in communicating benefit-risk decisions. Furthermore, the provision of a list of identified benefits and risks and visualizations would aim to facilitate communication by reducing the amount of text needed to convey these messages.

As a result of this study, future attempts to improve the quality of communication should include the following:

- A listing of benefits and risks, with justification for their roles in assessing the benefit-risk balance and the reasons for their inclusion or exclusion
- Valuing the identified benefits and risks
- Weighting (relative importance) of the identified benefits and risks
- Providing visualizations of the outcomes
- Utilizing guided discussions and structured questions such as deliberations on uncertainties, consistency of outcomes across studies and additional risks compared with a standard of care to illustrate key discussion points leading to benefit-risk decisions

Given that there are minimal differences among the existing formats of the reference agencies, it is timely to consider the feasibility of a universal template. The BR Template and BR Summary Template were based on the EMA reflection paper (European Medicines Agency, 2008) for the assessment of benefits and risks and to allow documentation of these considerations in support of the decision. Unlike the existing formats, the guided discussion, structure, listings of identified benefits and risks, application of values and weights and visualization of the BR Template serve to improve effective communication. Familiarity with a standard template and its presentation format will enhance the stakeholders' experience in seeking to understand the key messages. A universal framework for the assessment of benefits and risks will be required to bring focus to the agencies, which would then facilitate the implementation of a standard, universal documentation tool.

An eight-step universal benefit-risk framework has been developed that incorporates the existing frameworks of major regulatory agencies and those used by pharmaceutical companies (US Food and Drug Administration. Medical Review, 2012). Given that the BR Template and BR Summary Template was developed using the principles from this universal framework, there is now the opportunity to explore their universal use. However, as the basis for publicly available assessment reports, it would be prudent to seek more confirmative opinions from stakeholders on the feasibility and utility of such an initiative through further studies.

In the course of this research, some areas for improvement were identified for the BR Template and BR Summary Template. These included expanding the discussion on pharmacovigilance and RMP/REM, which would then align to the recent requirements for periodic benefit-risk evaluation report (PBRER) (European Medicines Agency, 2013b) and the emphasis on post-market activities. As stakeholders are increasingly seeking the acknowledgment of their opinions, there should also be dedicated and defined areas for inputs from the various stakeholders, particularly patients. These improvements may enable the BR Template to accommodate requirements in the post-marketing setting as well as functioning as a tool for product life cycle management. If used as a universal template, it could trace and document the evolution of the benefit-risk balance of a product and provide meaningful comparisons using valid baselines. Ultimately, this may translate to an increase in consistency, transparency and the quality decision-making.

### Conflict of interest statement

The authors declare that the research was conducted in the absence of any commercial or financial relationships that could be construed as a potential conflict of interest.
