# Supplementary material for: Strategy for communicating benefit-risk decisions: a comparison of regulatory agencies' publicly available documents
Source: Front Pharmacol. 2014 Dec 4;5:269. doi: 10.3389/fphar.2014.00269 (PMC4255503; doi:10.3389/fphar.2014.00269)
Supplement: Supplementary file 1 [file Table1.PDF]

Annex A

Comparison of sections of reference agencies' publicly available assessment reports with the BR Template

**BR Template**	**US FDA**		**EMA**	**Health Canada**	**TGA**
Content	Medical Review	Section 1<br><br>(Risk Benefit Assessment)	EPAR	SBD	AusPAR
**1 Background**					
1.1 Specify proposed therapeutic indication	Section 2	Analysis of condition	Section 1 – Scientific discussion	Not available	Section 1
1.2 Treatment modalities evaluated	Section 2	Analysis of condition	Section 1 – Scientific discussion	Not available	Section 1
1.3 Other current available treatment options not considered or evaluated	Section 2	Current treatment options	Section 1 – Scientific discussion	Not available	Section 1
1.4 Known risks with compounds of same therapeutic class	Section 2	Risk	Section 1 – Scientific discussion	Section 7 - Clinical	Section 1
1.5 Medical need	Section 2	Analysis of condition, Current treatment options	Section 1 – Scientific discussion	Section 2	Section 1
1.6 Aims of treatment and expected treatment size	Section 2	Analysis of condition, Current treatment options	Section 1 – Scientific discussion	Not available	Section 1

Comparison of sections of reference agencies' publicly available assessment reports with the BR Template (continued)

**BR Template**	**US FDA**		**EMA**	**Health Canada**	**TGA**
Content	Medical Review	Section 1 (Risk Benefit Assessment)	EPAR	SBD	AusPAR
**2 Overall Summary**					
2.1 Quality overall summary	Section 4	Not available	Section 2 – Quality aspects	Section 7 - Quality	Section 6 - Quality
2.2 Non-clinical overall summary	Section 4	Not available	Section 2 – Non-clinical aspects	Section 7 – Non-clinical	Section 6 – Non-clinical
2.2.1 Comments on relevant findings and potential implications/ investigations required	Section 4	Not available	Section 2 – Non-clinical aspects	Section 7 – Non-clinical	Section 3
2.2.2 Conclusions implicating benefit-risk assessment for humans	Section 4	Not available	Section 2 – Non-clinical aspects	Section 7 – Non-clinical	Section 3
2.3.1 Human pharmacology: Overall summary	Section 4	Not available	Section 2 – Clinical aspects	Section 7 – Clinical pharmacology	Section 4
2.3.2 Human pharmacology Conclusions	Section 6	Not available	Section 2 – Clinical aspects	Section 7 – Clinical pharmacology	Section 6 - Clinical
2.4.1 Clinical overall summary	Section 6	Benefit	Section 2 – Clinical efficacy	Section 7 – Clinical	Section 6 - Clinical

2.4.2 Clinical conclusions	Section 6	Benefit	Section 2 – Clinical efficacy	Section 7 – Clinical	Section 6 - Clinical
**3 Identified benefits and risks**					
3.1 Listing of all benefits, and justification for inclusion and exclusion	Not available	Not available	Not available	Not available	Not available
3.2 Listing of all risks, and justification for inclusion and exclusion	Not available	Not available	Not available	Not available	Not available

Comparison of sections of reference agencies' publicly available assessment reports with the BR Template (continued)

**BR Template**	**US FDA**		**EMA**	**Health Canada**	**TGA**
Content	Medical Review	Section 1<br><br>(Risk Benefit Assessment)	EPAR	SBD	AusPAR
**4 Benefit and Risk – Study information**					
4.1.1 – 4.1.9 Study details of benefit	Section 6	Benefit	Section 2 – Clinical efficacy	Section 7 – Clinical	Section 4
4.1.11 Discussion of consistency across all studies	Section 6	Benefit (Evidence and uncertainties)	Section 2 – Clinical efficacy	Section 7 – Clinical	Section 4
4.1.12 Discussion of evidence in relevant subgroups	Section 6	Benefit (Evidence and uncertainties)	Section 2 – Clinical efficacy	Section 7 – Clinical	Section 4
4.1.13 Discussion of confirmation by results of non-primary endpoint	Section 6	Benefit (Evidence and uncertainties)	Section 2 – Clinical efficacy	Section 7 – Clinical	Section 4
4.1.14 Discussion on patient reported outcomes	Section 6	Benefit (Evidence and uncertainties)	Section 2 – Clinical efficacy	Section 7 – Clinical	Section 4
4.1.15 Overall conclusion	Section 6	Benefit (Conclusions and reasons)	Section 2 – Clinical efficacy	Section 7 – Clinical	Section 4
4.2 Risks: Overall summary					

4.2.1 Overall incidence of adverse effects	Section 7	Risk	Section 2 – Clinical safety	Section 7 – Clinical	Section 4
4.2.2 Overall incidence of serious adverse effects		Risk	Section 2 – Clinical safety	Section 7 – Clinical	Section 4
4.2.3 Discontinuation rate due to AEs	Section 7	Risk	Section 2 – Clinical safety	Section 7 – Clinical	Section 4
4.2.4 Dose reduction rate due to AEs	Section 7	Risk	Section 2 – Clinical safety	Section 7 – Clinical	Section 4
4.3 Adverse effects	Section 7	Risk	Section 2 – Clinical safety	Section 7 – Clinical	Section 4
4.3.1 Details of AE	Section 7	Risk	Section 2 – Clinical safety	Section 7 – Clinical	Section 4

Comparison of sections of reference agencies' publicly available assessment reports with the BR Template (continued)

**BR Template**	**US FDA**		**EMA**	**Health Canada**	**TGA**
Content	Medical Review	Section 1<br>(Risk Benefit Assessment)	EPAR	SBD	AusPAR
4.4 Uncertainties (benefits and risks)					
4.4.1 Discussion on choice of dose, comparators and endpoints	Section 5	Evidence and uncertainties	Sections 2 & 3	Section 7 – Clinical	Section 6 – Risk-benefit analysis
4.4.2 Discussion on design, conduct and statistics	Section 5	Evidence and uncertainties	Sections 2 & 3	Section 7 – Clinical	Section 6 – Risk-benefit analysis

4.4.3 Discussion on validation of measurements and scales	Section 5	Evidence and uncertainties	Sections 2 & 3	Section 7 – Clinical	Section 6 – Risk-benefit analysis
4.4.4 Discussion on negative studies	Section 5 & 6	Evidence and uncertainties	Sections 2 & 3	Section 2 & 7 – Clinical	Section 6 – Risk-benefit analysis
4.4.5 Discussion of consistency across factors	Section 5 & 6	Evidence and uncertainties	Sections 2 & 3	Section 2 & 7 – Clinical	Section 6 – Risk-benefit analysis
4.4.6 Interactions with food/ drugs	Section 7	Risk	Section 2 – Clinical safety	Section 7 – Clinical	Section 4
4.4.7 Limitations of dataset regarding safety	Section 7	Risk (Evidence and uncertainties)	Section 2 – Clinical safety	Section 2 & 7 – Clinical	Section 6 – Risk-benefit analysis
4.4.8 Potential for off label use, overdose, abuse and misuse	Section 7	Risk (Evidence and uncertainties)	Section 2 – Clinical safety and pharmaco-vigilance	Section 2 & 7 – Clinical	Section 6 – Risk-benefit analysis
4.4.9 Risk with respect to standard of care	Section 7	Risk (Evidence and uncertainties)	Section 2 – Clinical safety	Section 3 – Benefit-risk assessment	Section 6 – Risk-benefit analysis
4.4.10 Comments on any other uncertainties	Section 5, 6 & 7	Evidence and uncertainties	Sections 2 & 3	Section 2	Section 6 – Risk-benefit analysis

Comparison of sections of reference agencies' publicly available assessment reports with the BR Template (continued)

BR Template	US FDA		EMA	Health Canada	TGA
Content	Medical Review	Section 1<br><br>(Risk Benefit	EPAR	SBD	AusPAR

		Assessment)			
**5 Benefit-risk Summary Table and Expert Judgment**					
5.1 Weighting and valuing of benefits	Not available	Not available	Not available	Not available	Not available
5.2 Weighting and valuing of risks	Not available	Not available	Not available	Not available	Not available
**6 Visualisation**	Not available	Not available	Not available	Not available	Not available
**7 Conclusions**					
7.1 Quality conclusions (pre-filled)	Section 4	Conclusions and reasons	Section 2	Section 7 - Quality	Section 6 – Quality
7.2 Non-clinical conclusions (pre-filled)	Section 4	Conclusions and reasons	Section 2	Section 7 – Non-clinical	Section 6 – Non-clinical
7.3 Human pharmacology conclusions (pre-filled)	Section 4	Conclusions and reasons	Section 2	Section 7 - Clinical	Section 6 – Clinical
7.4 Clinical conclusions (pre-filled)	Section 6	Conclusions and reasons	Section 2	Section 7 – Clinical	Section 6 - Clinical
7.4.1 For negative benefit-risk balance, discussion on the harm	Section 1 (Benefit-risk summary assessment)	Risk	Section 2	Section 2	Section 6 – Risk-benefit analysis
7.4.2 Discussion on evolution of the	Section 1 (Benefit-risk	Benefit-risk summary	Section 3	Section 2	Section 6 – Risk-benefit

benefit-risk balance	summary assessment)	assessment			analysis

Comparison of sections of reference agencies' publicly available assessment reports with the BR Template (continued)

BR Template	US FDA		EMA	Health Canada	TGA
Content	Medical Review	Section 1<br><br>(Risk Benefit Assessment)	EPAR	SBD	AusPAR
7.4.3 Discussion on outstanding issues and other significant information (hearings, advisories, patients, consumers, stakeholder inputs)	Section 1 (Benefit-risk summary assessment)	Benefit-risk summary assessment	Section 3	Section 2	Section 6 – Risk-benefit analysis
7.4.4 Discussion on pharmacovigilance plans and risk mitigation plans	Section 7, Section 1 (Benefit-risk summary assessment)	Risk management	Section 2 – Pharmaco-vigilance	Section 2 & 4	Section 6 – Risk management plan
7.4.5 Discussion on need for further studies	Section 6, Section 1 (Benefit-risk summary assessment)	Risk management	Sections 2 & 3	Section 2 & 4	Section 6 – Risk-benefit analysis
7.4.6 Any other information relevant to the benefit-risk decision	Section 1 (Benefit-risk summary	Benefit-risk summary assessment	Section 3	Section 2	Section 6 – Risk-benefit analysis

	assessment)				
7.4.7 Conclusion on the benefit-risk balance for proposed indication	Section 1 (Benefit-risk summary assessment)	Benefit-risk summary assessment	Sections 3 & 4	Section 2	Section 6 - Outcome
7.4.8 Recommendation indication	Section 1 (Benefit-risk summary assessment)	Benefit-risk summary assessment	Section 4	Section 1	Section 6 - Outcome

Annex B

Comparison of reference agencies' report templates with the BR Summary Template

**BR Summary Template**	**US FDA**	**EMA**	**Health Canada**	**TGA**
Content	Benefit-risk framework	EPAR – Executive Summary	SBD	AusPAR
**1.1 Background (Decision context)**				
1.1.1 Specify proposed therapeutic indication	Analysis of condition	Not available	Not available	Section 1
1.1.2 Treatment modalities evaluated	Current treatment options	Not available	Not available	Section 1
1.1.3 Medical need	Analysis of condition	Not available	Section 2	Section 1
**2.1 Overall summaries**		Not available		
2.1.1 Quality conclusions	Not available	Not available	Section 7 - Quality	Section 6
2.1.2 Non-clinical conclusions	Not available	Not available	Section 7 – Non-clinical	Section 6
2.1.3 Human pharmacology conclusions	Not available	Not available	Section 7 - Clinical	Section 6
2.1.4 Clinical conclusions	Benefit, Risk	Not available	Section 7 - Clinical	Section 6
**3.1 Identified benefits and risks**				
3.1.1 Listing of all benefits, and justification for	Not available	Not available	Not available	Not available

inclusion and exclusion				
3.1.2 Listing of all risks, and justification for inclusion and exclusion	Not available	Not available	Not available	Not available
**4.1 Clinical study summary**	Benefit	Not available	Section 7 - Clinical	Section 4
**5.1 Risks: Overall summary**	Risk	Not available	Section 7 - Clinical	Section 4
**6.1 Weighting and valuing of benefits and risks**	Not available	Not available	Not available	Not available

Comparison of reference agencies' report templates with the BR Summary Template (continued)

**BR Summary Template**	**US FDA**	**EMA**	**Health Canada**	**TGA**
Content	Benefit-risk framework	EPAR – Executive Summary	SBD	AusPAR
**7.1 Conclusion**				
7.1.1 For negative benefit-risk balance, discussion on the harms	Benefit-risk summary assessment	Not available	Section 2	Section 6
7.1.2 Discussion on evolution of the benefit-risk balance	Benefit-risk summary assessment	Not available	Section 2	Section 6
7.1.3 Discussion on outstanding issues and other significant information (hearings, advisories,	Benefit-risk summary assessment	Not available	Section 2	Section 6

patients, consumers, stakeholder inputs)				
7.1.4 Discussion on pharmacovigilance plans and risk mitigation plans	Risk Management	Not available	Section 2 & 4	Section 6
7.1.5 Discussion on need for further studies	Risk Management	Not available	Section 2 & 4	Section 6
7.1.6 Any other information relevant to the benefit-risk decision	Benefit-risk summary assessment	Not available	Section 2	Section 6
7.1.7 Conclusion on the benefit-risk balance for proposed indication	Benefit-risk summary assessment	Not available	Section 2	Section 6
7.1.8 Recommendation indication	Benefit-risk summary assessment	Not available	Section 1	Section 6

Annex C

US FDA Medical Review – Risk Benefit Assessment

Summary Template for the Benefit-Risk Assessment of Medicines

Participant(s):

HSA - Singapore

Compound Identifier(s):	Aflibercept / AVE005
Product name/ Brand name / Generic name:	Zaltrap
Active Ingredient(s)/ Strength(s)/ Dosage form:	Aflibercept, 100mg/4mL vial, 200mg/8ml vial
Proposed Indication:	Aflibercept is indicated in combination a FOLFIRI chemotherapy regimen for patients with metastatic colorectal cancer that is resistant to or has progressed after an oxaliplatin containing regimen.

BENEFIT RISK SUMMARY:

This section provides a summary of the key outcomes of Benefit Risk analysis undertaken.

Summary 1.1 Background (Decision Context):

Summary 1.1.1 Specify the proposed therapeutic indication

Metastatic colorectal carcinoma is a progressive disease with a fatal outcome. Median survival after diagnosis of the disease is approximately 22 months.

(Proposed indication not available from Risk Benefit Assessment but extracted from cover page of the Clinical Review)

Summary 1.1.2 Treatment modalities evaluated in this submission

In the adjuvant setting against placebo in patients with MCRC, background treatment with FOLFIRI.

Summary 1.1.3 Is this product for an unmet medical need? Please select: Yes

Reason: Please provide justification for your decision on the product fulfilling or not fulfilling an unmet medical need

Currently approved therapeutic options are reasonably well tolerated but provide limited effiacy (ie bevacizumab in the second line setting used in combination with oxalipatin median survival is 13 months compared to 10.8 mpnths in the chemotherapy/placebo arm., HR 0.75, 95% CI 0.63, 0.89). No monoclonal antibody targeting the VEGF pathway has been approved specifically in combination with FOLFIRI, a chemotherapy regimen commonly used in the US after progression following an oxaliplatin-containing regimen.

Summary 2.1 Overall Summaries:

Summary 2.1.1 Quality Conclusion:

If box ticked - No relevant findings for the clinical benefit-risk assessment ☐

If there are relevant findings please comment	Not available from Risk Benefit Assessment.<br><br>(However, required details are available from relevant sections of the Medical Review and Chemistry Review)

Summary 2.1.2 Non-Clinical Conclusion:

If box ticked - No relevant findings for the clinical benefit-risk assessment ☐

If there are relevant findings please comment	Not available from Risk Benefit Assessment.<br><br>(However, required details are available from relevant sections of the Medical Review and Pharmacology Review)

Summary 2.1.3 Human Phamacology Conclusion:

Only the important results and issues that have an impact on the benefit-risk balance should be described. In addition, unresolved issues or uncertainties should be identified and their impact on the balance assessment should be clearly stated. This includes Bioequivalence, Pharmacokinetic and Dynamic profile, as well as PK, & PD interactions, special populations, dose findings etc.

Not available from Risk Benefit Assessment.

(However, required details are available from relevant sections of the Medical Review and Clinical Pharmacology and Biopharmaceutics Review)

Summary 2.1.4 Clinical Conclusion:

Only the important results and issues that have an impact on the benefit-risk balance should be described. In addition, unresolved issues or uncertainties should be identified and their impact on the balance assessment should be clearly stated. This includes study design, dosage, population and comparators.

There are no drugs approved for the treatment of mCRC specifically in combination with FOLFIRI and no drugs have been approved for patients with prior bevacizumab treatment in the first-line setting. VELOUR was a well conducted study that showed that the addition of aflibercept to the FOLFIRI regimen resulted in a survival benefit, with a statistically significant log rank test with a p-value of 0.0032 (which met the pre specified efficacy boundary of 0.0466) and an estimated hazard ratio of 0.817 (95.34% CI: 0.713 to 0.937). The use of aflibercept resulted in a risk of death reduction of 18.3% when compared to placebo/FOLFIRI. Median overall survival (95.34% CI) in the placebo arm was 12.06 months (11.072 to 13.109), compared to 13.50 months (12.517 to 14.949) in the aflibercept arm. This benefit was supported by subgroup and sensitivity analyses, as well as the increased median PFS and response rates observed in the aflibercept arm. Furthermore, patients with prior exposure to bevacizumab appeared to benefit from treatment with aflibercept, although this benefit was of a smaller magnitude than in patients who have not been exposed bevacizumab (median OS for patients with prior exposure to bevicizumab in the placebo arm 11.7 months vs 12.5 months in the aflibercept arm; HR 0.86, 95% CI 0.67, 1.1).

The analysis of the database shows that aflibercept toxicity is within range (both in the type of events and the incidence rates) of bevacizumab, the only other VEGFR2 biologic inhibitor approved. Although the incidence rates of hypertension and proteinuria were higher than with bevacizumab, these differences may be a reflection of differences in monitoring as these toxicities are better understood. There are no new or unexpected safety signals when compared with bevacizumab.

BENEFIT RISK SUMMARY CONT:

Summary 3.1 Identified Benefits and Risks

Summary 3.1.1 Benefits documented

List all **benefits** of treatment for this indication as inferred in the submission	Please tick here if Benefit Identified by Reviewer but not by company	Please indicate which **benefits** you believe are justified to be included in the benefit risk assessment by ticking the box	Please explain your main reason **for inclusion or exclusion** of the benefit parameter
Overall survival	☐	☑	Not available from Risk Benefit Assessment.
Death reduction (HR)	☐	☑	Not available from Risk Benefit Assessment.
Progression-free survival	☐	☑	Not available from Risk Benefit Assessment.
Response rates	☐	☑	Not available from Risk Benefit Assessment.
	☐	☐	
	☐	☐	
	☐	☐	
	☐	☐	
	☐	☐	
	☐	☐	
	☐	☐	
	☐	☐	
	☐	☐	
	☐	☐	
	☐	☐	

BENEFIT RISK SUMMARY CONT:

Summary 3.1.2 Risks documented

List all **risks** of treatment for this indication as inferred in the submission	Please tick here if Risk Identified by Reviewer but not by company	Please indicate which **risks** you believe are justified to be included in the benefit risk assessment by ticking the box	Please explain your main reason **for inclusion or exclusion** of the risk parameter
Hypertension	☐	☑	Adverse events of special interest
Proteinuria	☐	☑	Adverse events of special interest
Arterial thrombotic events	☐	☑	Adverse events of special interest
Haemorrhage	☐	☑	Adverse events of special interest
Fistula	☐	☑	Adverse events of special interest
GI perforation	☐	☑	Adverse events of special interest
Leukopenia, neutropenia, thrombocytopenia	☐	☑	Adverse events of special interest
Reversible posterior leukoencephalopathy	☐	☑	Known treatment effect with anticancers and targeted VEGFR inhibitors
	☐	☐	
	☐	☐	
	☐	☐	
	☐	☐	
	☐	☐	
	☐	☐	
	☐	☐	

Summary 4.1 Clinical Study Summary

Study Ref. Type	Study Design (N)(duration) R, C, DB, OL (N=)(weeks/months) ·Non-inferiority/Superiority/ Observational study ·State primary objective ·State primary efficacy parameter	Treatment ·Treatment arm Active (name, dose, freq, duration) ·Comparator arm Placebo / Active (name, dose, freq, duration)	Conclusion ·Results of primary efficacy parameter ·Results of other relevant efficacy endpoints ·Conclusion of study (outcomes, strength of study, weight of evidence, and clinical significance)	
VELOUR<br>Pivotal	Superiority design<br>Primary objective of comparing efficacy.<br>Primary efficacy parameter based on death / survival.	Treatment: Aflibercept, IV, 4mg/kg over 1 hour, in combination with FOLFIRI, every 2 weeks.<br>Comparator: Placebo, IV, over 1 hour, combination with FOLFIRI, every 2 weeks.	Median OS: 13.50 months vs 12.06 months in placebo.<br>Median OS reduced by 1.44 months. Risk of death reduced by 18.3% (HR 0.817, CI 0.713 - 0.937).<br>PFS: 6.9 months vs 4.7 months placebo, HR 0.756<br>ORR: 20% vs 11% in placebo	-
				-
				-
				-
				-

Legend
R: Randomised **C:** Controlled **DB:** Double blinded **OL:** Open label **N:** Number of subjects

Click to add a study +

Summary 5.1 RISKS: Overall Summary

Table of pooled overall incidence of events can be added below

Adobe **Acrobat** users can click here to attach a file: Attach a file *(Note: this will not activate in Adobe* ***Reader****)*

Click in the space below to upload an image: (jpeg, gif, png): *(Available to both Adobe Reader and Acrobat users)*

No tables or figures available

BENEFIT RISK SUMMARY CONT:

Summary 6.1 Weights and values

Benefits	Relative Importance (weighting)	Valuing the options			Comment on strength and uncertainty of benefit
		Investigated product	Comparator	Placebo	
Overall survival	Not available from risk benefit assessment.	13.50 months		12.06 months	Not available from Risk Benefit Assessment.
Death reduction (HR)	Not available from risk benefit assessment.	0.817			Not available from Risk Benefit Assessment.
Progression-free survival	Not available from risk benefit assessment.	6.9 months		4.7 months	Not available from Risk Benefit Assessment.
Response rates	Not available from risk benefit assessment.	20%		11%	Not available from Risk Benefit Assessment.

Please describe methodology used for assessing relative importance: eg Ranking or point allocation and also what is has been used in relation to valuing the options e.g. % change, Number of patients, etc

Not available from Risk Benefit Assessment.

Please describe methodology used for assessing relative importance: eg Ranking or point allocation and also what is has been used in relation to valuing the options e.g. % change, Number of patients, etc

Not available in Executive Summary

BENEFIT RISK SUMMARY CONT:

Risks	Relative Importance (weighting)	Valuing the options Investigated product	Comparator	Placebo	Comment on strength and uncertainty of each risk	Was the value or weight of this risk altered or mitigated by the ability to control the use of the medicine once on the market?
Hypertension	Not available from Risk Benefit Assessment.	41%		11%	Not available from Risk Benefit Assessment.	Through product labeling and use by oncologists.
Proteinuria	Not available from Risk Benefit Assessment.	62%		41%	Not available from Risk Benefit Assessment.	Through product labeling and use by oncologists.
Arterial thrombotic events	Not available from Risk Benefit Assessment.	2.6%		1.65%	Not available from Risk Benefit Assessment.	Through product labeling and use by oncologists.
Haemorrhage	Not available from Risk Benefit Assessment.	38%		19%	Not available from Risk Benefit Assessment.	Through product labeling and use by oncologists.
Fistula	Not available from Risk Benefit Assessment.	9 patients		3 patients	Not available from Risk Benefit Assessment.	Through product labeling and use by oncologists.
GI perforation	Not available from Risk Benefit Assessment.	3 patients		3 patients	Not available from Risk Benefit Assessment.	Through product labeling and use by oncologists.
Leukopenia, neutropenia, thrombocytopenia	Not available from Risk Benefit Assessment.	16%, 36%, 3%		12%, 30%, 2%	Not available from Risk Benefit Assessment.	Through product labeling and use by oncologists.
Reversible posterior leukoencephalopathy	Not available from Risk Benefit Assessment.	0		0	Not available from Risk Benefit Assessment.	Through product labeling and use by oncologists.

Please describe methodology used for assessing relative importance: eg Ranking or point allocation and also what is has been used in relation to valuing the options e.g. % change, Number of patients, etc

Not available from Risk Benefit Assessment.

Please describe methodology used for assessing relative importance: eg Ranking or point allocation and also what is has been used in relation to valuing the options e.g. % change, Number of patients, etc

Not available in Executive Summary

BENEFIT RISK SUMMARY CONT:

Summary 7.1 Conclusion

**Summary 7.1.1** If the benefit-risk balance is assessed to be negative, describe the harm (e.g. in terms of lack of efficacy, toxicity) that the drug may cause if used in the proposed indication
Not applicable.
**Summary 7.1.2** Describe how the benefit-risk balance is expected to evolve over time (e.g. when late side effects emerge or long-term efficacy decreases)
Not available from Risk Benefit Assessment.<br><br>(However, required details are available from sections on Review of Efficacy and Review of Safety)
**Summary 7.1.3** Describe outstanding issues, and other significant information eg, submission of additional reports by the company to address those issues, hearings and advisory group recommendations, information from other jurisdictions (eg advisory committees, scientific experts, patients, consumers, consumer advocates and other stakeholders)
Not available from Risk Benefit Assessment.<br><br>(However, required details are available from sections on Review of Efficacy and Review of Safety)
**Summary 7.1.4** Make reference to the evaluation of the pharmacovigilance plan and risk minimization plan if any. Describe any communication or particularly significant information to the medical profession, patients or the public that is required. Describe restrictions to product availability or usage
A post marketing commitment (PMC) is proposed to obtain the data of study NCT0062241, a Phase 1 study of aflibercept in children with refractory solid tumors. This study was conducted under the NCI aflibercept IND 100137 by the Children's Oncology Group (protocol COGADVL0714) and it is complete. The purpose of this PMC is to analyze this data to include it in the pediatric section of the Zaltrap label.
**Summary 7.1.5** Describe the need for further studies (e.g. the need for studies to improve the benefit-risk balance with further optimization studies, the need for intensive additional follow up measures or specific obligations, and the need for further development including any paediatric development plans.
A post marketing commitment (PMC) is proposed to obtain the data of study NCT0062241, a Phase 1 study of aflibercept in children with refractory solid tumors. This study was conducted under the NCI aflibercept IND 100137 by the Children's Oncology Group (protocol COGADVL0714) and it is complete. The purpose of this PMC is to analyze this data to include it in the pediatric section of the Zaltrap label.

Summary 7.1.6 Please provide any other information considered by the agency relevant to the benefit risk decision that is not covered elsewhere in the proforma.

The risks of aflibercept use in the treatment of MCRC whose disease had progressed after a first-line treatment with an xoaplatin-containing regimen will be managed through product labeling. The risks are also managed in that this drug will be administered by oncologists who have specific training in the administration of anti-neoplastic drugs and in the management of toxicities related to these drugs.

Summary 7.1.7 Please provide a clear conclusion on the benefit-risk being positive or not for the proposed indication.

In summary, the approval is recommended based on a prolongation of overall survival with an acceptable toxicity profile (toxicity in this setting refers to the additional toxicity of aflibercept when added to the FOLFIRI regiment), for which the oncology community has experience in its management. The study effects were supported by secondary endpoints including PFS and ORR.

Summary 7.1.8 Please provide the indication recommended following the outcome of the benefit-risk balance.

Approval is recommended for the use of aflibercept in combination with the FOLFIRI regimen for the treatment of patients with metastatic colorectal carcinoma that is resistant to or has progressed after an oxaliplatin-containing regimen.

Reviewers Name: Sandra J Casak

Signature:

Date: 28 March 2012

Manager sign-off or Peer review

Reviewers Name: Steven J Lemery

Signature:

Date: 28 March 2012

EPAR – Executive Summary

Summary Template for the Benefit-Risk Assessment of Medicines

Participant(s):

HSA - Singapore

Compound Identifier(s):	Aflibercept
Product name/ Brand name / Generic name:	Zaltrap
Active Ingredient(s)/ Strength(s)/ Dosage form:	Aflibercept, 100mg/4mL vial, 200mg/8mL vial
Proposed Indication:	Zaltrap in combination with irinotecan/5-fluoouracil/ folinic acid (FOLFIRI) chemotherapy is indicated in adults with metastatic colorectal cancer (MCRC) that is resistant to or has progressed ater an oxaliplatin-containing regimen.

BENEFIT RISK SUMMARY:

This section provides a summary of the key outcomes of Benefit Risk analysis undertaken.

Summary 1.1 Background (Decision Context):

Summary 1.1.1 Specify the proposed therapeutic indication

Colorectal cancer (CRC) is one of the most common cancers in both men and women, and the second most common cause of cancer mortality in Europe. Significant advances in the treatment of metastatic CRC have been made during the last 25 years with the introduction of chemotherapy agents. Current therapies used in clinical practice for first and second line treatment of metastatic CRC include irinotecan or oxaliplatin, each in combination with bolus and infusional 5FU/ LV. Standard second-line treatments for metastatic CRC have also evolved to include the addition of targeted biologic therapies such as bevacizumab, cetuximab and panitumumab. Despite these advances, the prognosis of patients with metastatic CRC undergoing second-line treatment is poor and the expected median overall survival is only approximately one year.

In November 2012, the European Medicines Agency's Committee for Medicinal Products for Human Use (CHMP) recommended the authorisation of aflibercept (Zaltrap) in combination with irinotecan/5-fluorouracil/folinic acid (FOLFIRI) chemotherapy in the treatment of adults with metastatic colorectal cancer (MCRC) that is resistant to or has progressed after an oxaliplatin-containing regimen. The recommended dose of aflibercept, administered as an intravenous infusion over 1 hour, is 4 mg/kg of body weight, followed by the FOLFIRI regimen. This is considered as one treatment cycle. The treatment cycle is repeated every 2 weeks.

Summary 1.1.2 Treatment modalities evaluated in this submission

The demonstration of clinical benefit for aflibercept was based on a single randomised, double-blind controlled trial of aflibercept versus placebo in MCRC patients being treated with FOLFIRI after failure of an oxaliplatin based regimen (EFC10262- VELOUR).

Summary 1.1.3 Is this product for an unmet medical need? Please select: Yes

Reason: Please provide justification for your decision on the product fulfilling or not fulfilling an unmet medical need

Despite these advances, the prognosis of patients with metastatic CRC undergoing second-line treatment is poor and the expected median overall survival is only approximately one year.

Summary 2.1 Overall Summaries:

Summary 2.1.1 Quality Conclusion:

If box ticked - No relevant findings for the clinical benefit-risk assessment ☐

If there are relevant findings please comment	Not available from Executive Summary<br><br>(However, required details are available from Quality aspects discussion in the EPAR)

Summary 2.1.2 Non-Clinical Conclusion:

If box ticked - No relevant findings for the clinical benefit-risk assessment ☐

If there are relevant findings please comment	Not available in Executive Summary<br><br>(However, required details are available from Non-clinical aspects discussion in the EPAR)

Summary 2.1.3 Human Phamacology Conclusion:

Only the important results and issues that have an impact on the benefit-risk balance should be described. In addition, unresolved issues or uncertainties should be identified and their impact on the balance assessment should be clearly stated. This includes Bioequivalence, Pharmacokinetic and Dynamic profile, as well as PK, & PD interactions, special populations, dose findings etc.

Not available in Executive Summary

(However, required details are available from Clinical aspects discussion in the EPAR)

Summary 2.1.4 Clinical Conclusion**:**

Only the important results and issues that have an impact on the benefit-risk balance should be described. In addition, unresolved issues or uncertainties should be identified and their impact on the balance assessment should be clearly stated. This includes study design, dosage, population and comparators.

The demonstration of clinical benefit for aflibercept was based on a single randomised, double-blind controlled trial of aflibercept versus placebo in MCRC patients being treated with FOLFIRI after failure of an oxaliplatin based regimen (EFC10262- VELOUR). In this trial, the risk of death associated with aflibercept was reduced by 18% compared to that observed in the control group. Aflibercept was associated with an improvement of 2.23 months in duration of median progression-free survival and of 9% in objective response rate.

The trial also included a subgroup of patients whose disease had progressed after treatment with bevacizumab. In this subgroup analysis, a trend towards a favourable effect on overall survival was observed for aflibercept, but no definitive conclusions could be drawn.

(No safety specific summary available from Executive Summary)

<u>BENEFIT RISK SUMMARY CONT:</u>

<u>Summary 3.1 Identified Benefits and Risks</u>

Summary 3.1.1 Benefits documented

List all **benefits** of treatment for this indication as inferred in the submission	Please tick here if Benefit Identified by Reviewer but not by company	Please indicate which **benefits** you believe are justified to be included in the benefit risk assessment by ticking the box	Please explain your main reason **for inclusion or exclusion** of the benefit parameter
Overall survival	☐	☑	Not available in Executive Summary
Risk of death (HR)	☐	☑	Not available in Executive Summary
Progression-free survival	☐	☑	Not available in Executive Summary
Objective response rate	☐	☑	Not available in Executive Summary
	☐	☐	
	☐	☐	
	☐	☐	
	☐	☐	
	☐	☐	
	☐	☐	
	☐	☐	
	☐	☐	
	☐	☐	
	☐	☐	
	☐	☐	

BENEFIT RISK SUMMARY CONT:

Summary 3.1.2 Risks documented

List all **risks** of treatment for this indication as inferred in the submission	Please tick here if Risk Identified by Reviewer but not by company	Please indicate which **risks** you believe are justified to be included in the benefit risk assessment by ticking the box	Please explain your main reason **for inclusion or exclusion** of the risk parameter
Hypertension	☐	☑	Not available in Executive Summary
Haemorrhage	☐	☑	Not available in Executive Summary
Fistulae	☐	☑	Not available in Executive Summary
Diarrhea	☐	☑	Not available in Executive Summary
Neutropenia	☐	☑	Not available in Executive Summary
Stomatitis	☐	☑	Not available in Executive Summary
Asthenic conditions	☐	☑	Not available in Executive Summary
Ulceration	☐	☑	Not available in Executive Summary
Dehydration	☐	☑	Not available in Executive Summary
Infections and infestations	☐	☑	Not available in Executive Summary
Weight decrease	☐	☑	Not available in Executive Summary
GI disorders	☐	☑	Not available in Executive Summary
	☐	☐	
	☐	☐	
	☐	☐	

Summary 4.1 Clinical Study Summary

Study Ref. Type	Study Design (N)(duration) R, C, DB, OL (N=)(weeks/months)<br>·Non-inferiority/Superiority/ Observational study<br>·State primary objective<br>·State primary efficacy parameter	Treatment<br>·Treatment arm Active (name, dose, freq, duration)<br>·Comparator arm Placebo / Active (name, dose, freq, duration)	Conclusion<br>·Results of primary efficacy parameter<br>·Results of other relevant efficacy endpoints<br>·Conclusion of study (outcomes, strength of study, weight of evidence, and clinical significance)	
VELOUR<br>Pivotal	Superiority design. Primary objective is efficacy in MCRC patients after oxaplatin-regimen failure. Primary efficacy endpoint is median overall survival.	Treatment: Aflibercept, IV, 4mg/kg over 1 hour, followed by FOLFIRI. Repeat cycle every 2 weeks.<br>Comparator: Placebo, IV, over 1 hour, followed by FOLFIRI. Repeat cycle every 2 weeks.	Median OS: 13.5 months vs 12.1 months in placebo. Median OS reduced by 1.44 months. Risk of death reduced by 18% (HR 0.817, CI 0.713 - 0.937, p=0.0032).<br>PFS: increased by 2.23 months<br>ORR: increased by 9%	-
				-
				-
				-
				-
Legend<br>**R:** Randomised **C:** Controlled **DB:** Double blinded **OL:** Open label **N:** Number of subjects			Click to add a study	+

Summary 5.1 RISKS: Overall Summary

Table of pooled overall incidence of events can be added below

Adobe **Acrobat** users can click here to attach a file: Attach a file *(Note: this will not activate in Adobe **Reader**)*

Click in the space below to upload an image: (jpeg, gif, png): *(Available to both Adobe Reader and Acrobat users)*

No tables or figures available

BENEFIT RISK SUMMARY CONT:

Summary 6.1 Weights and values

Benefits	Relative Importance (weighting)	Valuing the options			Comment on strength and uncertainty of benefit
		Investigated product	Comparator	Placebo	
Overall survival	Not available in Executive Summary	13.5 months		12.1 months	Not available in Executive Summary
Risk of death (HR)	Not available in Executive Summary	0.817			Not available in Executive Summary
Progression-free survival	Not available in Executive Summary	Not available from executive summary; improvement by 2.23 months over placebo		Not available from executive summary	Not available in Executive Summary
Objective response rate	Not available in Executive Summary	19.8%		11.1%	Not available in Executive Summary

Please describe methodology used for assessing relative importance: eg Ranking or point allocation and also what is has been used in relation to valuing the options e.g. % change, Number of patients, etc

Not available in Executive Summary

BENEFIT RISK SUMMARY CONT:

Risks	Relative Importance (weighting)	Valuing the options Investigated product	Comparator	Placebo	Comment on strength and uncertainty of each risk	Was the value or weight of this risk altered or mitigated by the ability to control the use of the medicine once on the market?
Hypertension	Not available in Executive Summary				Not available in Executive Summary	Not available in Executive Summary
Haemorrhage	Not available in Executive Summary				Not available in Executive Summary	Not available in Executive Summary
Fistulae	Not available in Executive Summary				Not available in Executive Summary	Not available in Executive Summary
Diarrhea	Not available in Executive Summary				Not available in Executive Summary	Not available in Executive Summary
Neutropenia	Not available in Executive Summary				Not available in Executive Summary	Not available in Executive Summary
Stomatitis	Not available in Executive Summary				Not available in Executive Summary	Not available in Executive Summary
Asthenic conditions	Not available in Executive Summary				Not available in Executive Summary	Not available in Executive Summary
Ulceration	Not available in Executive Summary				Not available in Executive Summary	Not available in Executive Summary
Dehydration	Not available in Executive Summary				Not available in Executive Summary	Not available in Executive Summary
Infections and infestations	Not available in Executive Summary	11.3		6.3	Not available in Executive Summary	Not available in Executive Summary
Weight decrease	Not available in Executive Summary				Not available in Executive Summary	Not available in Executive Summary
GI disorders	Not available in Executive Summary	20		11	Not available in Executive Summary	Not available in Executive Summary

Please describe methodology used for assessing relative importance: eg Ranking or point allocation and also what is has been used in relation to valuing the options e.g. % change, Number of patients, etc

Not available in Executive Summary

BENEFIT RISK SUMMARY CONT:

Summary 7.1 Conclusion

**Summary 7.1.1** If the benefit-risk balance is assessed to be negative, describe the harm (e.g. in terms of lack of efficacy, toxicity) that the drug may cause if used in the proposed indication
Not applicable.
**Summary 7.1.2** Describe how the benefit-risk balance is expected to evolve over time (e.g. when late side effects emerge or long-term efficacy decreases)
In terms of balance of benefits and risks, the overall toxicity of aflibercept in the studied combination regimen was considered significant, not always manageable, and in some patients ultimately leading to termination also of the chemotherapy.<br>However, despite this toxicity, there was still a small but clinically relevant survival advantage of 1.44 months (median). Thus, the benefits associated with aflibercept were considered to outweigh the risks.
**Summary 7.1.3** Describe outstanding issues, and other significant information eg, submission of additional reports by the company to address those issues, hearings and advisory group recommendations, information from other jurisdictions (eg advisory committees, scientific experts, patients, consumers, consumer advocates and other stakeholders)
Not available in Executive Summary<br><br>(However, required details are available from the Benefit-risk Balance section in the EPAR)
**Summary 7.1.4** Make reference to the evaluation of the pharmacovigilance plan and risk minimization plan if any. Describe any communication or particularly significant information to the medical profession, patients or the public that is required. Describe restrictions to product availability or usage
Not available in Executive Summary<br><br>(However, required details are available from Pharmacovigilance discussion in the EPAR)
**Summary 7.1.5** Describe the need for further studies (e.g. the need for studies to improve the benefit-risk balance with further optimization studies, the need for intensive additional follow up measures or specific obligations, and the need for further development including any paediatric development plans.
In order to optimise benefit–risk balance, it is essential to identify the proper target population for therapy. This might be possible to accomplish through the judicious use of biomarkers in all phases of clinical drug development. However, no validated predictive serum or plasma biomarkers have been identified during the development of aflibercept that correlate with treatment outcomes. Thus, the CHMP has requested to the applicant company to analyse plasma and tissue samples from the available trials, with the primary aim to identify biomarkers to allow better selection of the population likely to experience a beneficial effect following treatment with aflibercept.

Summary 7.1.6 Please provide any other information considered by the agency relevant to the benefit risk decision that is not covered elsewhere in the proforma.

Not available in Executive Summary

(However, required details are available from the User conclusion discussion and Benefit-risk Balance section in the EPAR)

Summary 7.1.7 Please provide a clear conclusion on the benefit-risk being positive or not for the proposed indication.

In terms of balance of benefits and risks, the overall toxicity of aflibercept in the studied combination regimen was considered significant, not always manageable, and in some patients ultimately leading to termination also of the chemotherapy. However, despite this toxicity, there was still a small but clinically relevant survival advantage of 1.44 months (median). Thus, the benefits associated with aflibercept were considered to outweigh the risks.

Summary 7.1.8 Please provide the indication recommended following the outcome of the benefit-risk balance.

In November 2012, the European Medicines Agency's Committee for Medicinal Products for Human Use (CHMP) recommended the authorisation of aflibercept (Zaltrap) in combination with irinotecan/5-fluorouracil/folinic acid (FOLFIRI) chemotherapy in the treatment of adults with metastatic colorectal cancer (MCRC) that is resistant to or has progressed after an oxaliplatin-containing regimen. The recommended dose of aflibercept, administered as an intravenous infusion over 1 hour, is 4 mg/kg of body weight, followed by the FOLFIRI regimen. This is considered as one treatment cycle. The treatment cycle is repeated every 2 weeks.

Reviewers Name: Kristina Dunder and Daniela Melchiorri (information extracted from EPAR)

Signature:

Date: 30 October 2012

Manager sign-off or Peer review

Reviewers Name: Not available

Signature:

Date: Not available

AusPAR – Overall Conclusion and Risk Benefit Assessment

Summary Template for the Benefit-Risk Assessment of Medicines

Participant(s):

HSA - Singapore

Compound Identifier(s):	Aflibercept rch
Product name/ Brand name / Generic name:	Zaltrap, Aflitiv, Lidaveg
Active Ingredient(s)/ Strength(s)/ Dosage form:	Aflibercept 100mg/4ml, 200mg/8ml
Proposed Indication:	Aflibercept in combination with irinotecan-fluoropyrimidine-based chemotherapy is indicated in adults with metastatic colorectal cancer (MCRC) previously treated with an oxaliplatin-containing regimen.

BENEFIT RISK SUMMARY:

This section provides a summary of the key outcomes of Benefit Risk analysis undertaken.

Summary 1.1 Background (Decision Context):

Summary 1.1.1 Specify the proposed therapeutic indication

The sponsor aims to register the product for the indication: "aflibercept in combination with irinotecan-fluoropyrimidine-based chemotherapy is indicated in adults with metastatic colorectal cancer (MCRC) previously treated with an oxaliplatin-containing regimen". Thus, the proposal is for second line use, alongside other agents, in the setting of metastatic colorectal cancer.

The VEGF pathway plays an important role in angiogenesis and is considered important in the pathological angiogenesis seen in MCRC.

Three biologicals are currently approved for (at least) second line treatment: bevacizumab (anti-VEGF), cetuximab (anti-EGFR) and panitumumab (anti-EGFR). The sponsor states that none of these has shown a statistically significant improvement in OS in combination with FOLFIRI when compared to FOLFIRI alone.

Summary 1.1.2 Treatment modalities evaluated in this submission

Three biologicals are currently approved for (at least) second line treatment: bevacizumab (anti-VEGF), cetuximab (anti-EGFR) and panitumumab (anti-EGFR). The sponsor states that none of these has shown a statistically significant improvement in OS in combination with FOLFIRI when compared to FOLFIRI alone.

Summary 1.1.3 Is this product for an unmet medical need? Please select: Yes

**Reason:** Please provide justification for your decision on the product fulfilling or not fulfilling an unmet medical need	In conclusion, aflibercept plus FOLFIRI is the only regimen to demonstrate a significant and clinically meaningful OS and PFS advantage in MCRC, with a assessment, in an indication with a high unmet medical need where patients have few therapeutic options.<br><br>One pivotal efficacy and safety study was submitted. This was Study EFC10262 (VELOUR), a randomised, double-blind, placebo-controlled trial.

Summary 2.1 Overall Summaries:

Summary 2.1.1 Quality Conclusion:

If box ticked - No relevant findings for the clinical benefit-risk assessment ☑

If there are relevant findings please comment	There were no objections to registration from a biochemistry / molecular biology perspective.

Summary 2.1.2 Non-Clinical Conclusion:

If box ticked - No relevant findings for the clinical benefit-risk assessment ☑

If there are relevant findings please comment	Major targets for toxicity in monkeys included: nasal cavity; bone (for example, vertebral exostoses associated with kyphosis), kidneys (e.g. increased glomerular mesangial matrix; glomerulopathy with tubular dilatation and cast formation), adrenals and ovary (for example, reversible reduction in maturing follicles, with profound reduction in ovarian hormones).<br><br>There were no objections to registration from the nonclinical evaluator.

Summary 2.1.3 Human Phamacology Conclusion:

Only the important results and issues that have an impact on the benefit-risk balance should be described. In addition, unresolved issues or uncertainties should be identified and their impact on the balance assessment should be clearly stated. This includes Bioequivalence, Pharmacokinetic and Dynamic profile, as well as PK, & PD interactions, special populations, dose findings etc.

Pharmacokinetics:

The following specific issues were identified:

· No formal PK study in hepatic impairment was submitted. Results from VELOUR suggested mild and moderate impairment did not affect free aflibercept PK. There were limited data for moderate and severe impairment.

· No formal PK study in renal impairment was submitted. In POH0625, free aflibercept clearance fell with increasing renal impairment, but systemic exposure was not markedly affected. The sponsor maintains that "since high molecular weight proteins are not cleared by [renal elimination], renal elimination of aflibercept is expected to be minimal". Perhaps a confounding factor influences clearance and is associated with renal function. No patients in VELOUR had severe renal impairment.

· In VELOUR, exposure to free aflibercept increased with body weight, and clearance decreased, despite dosing according to weight. AUC was 29% higher in >100 kg patients than in 50-100 kg patients (analysis of AEs by weight raised no concerns).

· With combination treatments (that is, aflibercept plus other agents), relative to treatment with aflibercept alone, there was higher exposure to bound aflibercept.

The effect of concomitant aflibercept on PK of multiple other anti-cancer agents was studied using historical comparison. There was a suggestion that clearance of gemcitabine was lower with concomitant aflibercept, though this is an exploratory finding.

Pharmacodynamics:

Studies PDY6655 (blood pressure), PDY6656 (blood pressure) and TES10897 (QT interval) provided PD data. The evaluator discounted PDY6656 as it was not placebo-controlled and lacked a 4 mg/kg arm.

Summary 2.1.4 Clinical Conclusion:

Only the important results and issues that have an impact on the benefit-risk balance should be described. In addition, unresolved issues or uncertainties should be identified and their impact on the balance assessment should be clearly stated. This includes study design, dosage, population and comparators.

The 4 mg/kg dose used in VELOUR was determined in a Phase I study (TCD6118) of 2, 4, 5 and 6 mg/kg doses in patients with solid tumours. The evaluator considered that the optimum dose has not been characterised.

The clinical evaluator has recommended rejection of the application. The clinical evaluator states that "results are considered to be clinically insignificant, based on the survival parameters used to calculate the sample size". Basis found only in main clinical report and not in section of overall conclusion.

The Delegate considers that OS results are statistically and clinically significant, in the context of available treatments. Sample size criteria are used to design an adequately powered study. In the Delegate's view, the minimum clinically significant difference in OS may differ from the parameters used to calculate sample size.

BENEFIT RISK SUMMARY CONT:

Summary 3.1 Identified Benefits and Risks

Summary 3.1.1 Benefits documented

List all **benefits** of treatment for this indication as inferred in the submission	Please tick here if Benefit Identified by Reviewer but not by company	Please indicate which **benefits** you believe are justified to be included in the benefit risk assessment by ticking the box	Please explain your main reason **for inclusion or exclusion** of the benefit parameter
Overall survival	☐	☑	Not available in Overall conclusion and risk/benefit assessment
Progression-free survival	☐	☑	Not available in Overall conclusion and risk/benefit assessment
Objective response rate	☐	☑	Not available in Overall conclusion and risk/benefit assessment
Quality of life	☑	☐	Not available in Overall conclusion and risk/benefit assessment
	☐	☐	
	☐	☐	
	☐	☐	
	☐	☐	
	☐	☐	
	☐	☐	
	☐	☐	
	☐	☐	
	☐	☐	
	☐	☐	
	☐	☐	

BENEFIT RISK SUMMARY CONT:

Summary 3.1.2 Risks documented

List all **risks** of treatment for this indication as inferred in the submission	Please tick here if Risk Identified by Reviewer but not by company	Please indicate which **risks** you believe are justified to be included in the benefit risk assessment by ticking the box	Please explain your main reason **for inclusion or exclusion** of the risk parameter
Deaths	☐	☑	Not available in Overall conclusion and risk/benefit assessment
Hypertension	☐	☑	Hypertension is an anti-VEGF class effect.
QT prolongation	☐	☑	Not available in Overall conclusion and risk/benefit assessment
Haemorrhage	☐	☑	Haemorrhage is an anti-VEGF class effect.
Proteinuria	☐	☑	Proteinuria is a class effect of agents targeting the VEGF pathway.
Diarrhea and stomatitis	☐	☑	Not available in Overall conclusion and risk/benefit assessment
Fistulae and gastrointestinal perforation	☐	☑	Not available in Overall conclusion and risk/benefit assessment
Thromboembolism	☐	☑	Not available in Overall conclusion and risk/benefit assessment
Blood cell counts	☐	☑	Not available in Overall conclusion and risk/benefit assessment
Hepatic and renal toxicity	☐	☑	Not available in Overall conclusion and risk/benefit assessment
Dysphonia	☐	☑	Dysphonia was described as due to VEGF blockade.
Immunogenicity	☐	☑	Not available in Overall conclusion and risk/benefit assessment
	☐	☐	
	☐	☐	
	☐	☐	

Summary 4.1 Clinical Study Summary

Study Ref.<br>Type	Study Design<br>(N)(duration)<br>R, C, DB, OL<br>(N=)(weeks/months)<br><br>·Non-inferiority/Superiority/ Observational study<br>·State primary objective<br>·State primary efficacy parameter	Treatment<br><br>·Treatment arm<br>Active (name, dose, freq, duration)<br>·Comparator arm<br>Placebo / Active (name, dose, freq, duration)	Conclusion<br>·Results of primary efficacy parameter<br><br>·Results of other relevant efficacy endpoints<br><br>·Conclusion of study (outcomes, strength of study, weight of evidence, and clinical significance)	
VELOUR<br>Pivotal	VELOUR was a Phase III, randomised, double-blind, placebo-controlled trial in adult patients with MCRC being treated with irinotecan / 5-FU / leucovorin (FOLFIRI) following disease progression while on or after completion of treatment with an oxaliplatin-based regimen. The primary endpoint was OS.	Treatment: Aflibercept, IV, 4mg/kg over 1 hour, followed immediately with FOLFIRI, and repeated every two weeks.<br>Comparator: Placebo, IV, 4mg/kg over 1 hour, followed immediately with FOLFIRI, and repeated every two weeks.	Median OS in the placebo arm was 12.1 months and in the aflibercept arm 13.5 months (HR 0.817, 95.34% CI 0.713-0.937). Overall survival may, in general terms, be influenced by effective treatments received after study drug discontinuation. Post-study anti-cancer treatment was balanced across arms.<br><br>Progression free survival was generally based on independent, blinded third-party review by the IRC. Median PFS was 4.7 months for placebo versus 6.9 months for aflibercept (HR 0.758, 99.99% CI 0.578 to 0.995).<br><br>ORR was analysed in the "evaluable patient population" with IRC assessment. An overall response was seen in 11.1% (placebo) versus 19.8% (aflibercept), although the only two patients with a complete response were in the placebo arm.	-
				-
				-
				-

Summary 5.1 RISKS: Overall Summary

Table of pooled overall incidence of events can be added below

Adobe **Acrobat** users can click here to attach a file: Attach a file *(Note: this will not activate in Adobe **Reader**)*

Click in the space below to upload an image: (jpeg, gif, png): *(Available to both Adobe Reader and Acrobat users)*

n(%)	Placebo/Folfiri (N=605)	Aflibercept/Folfiri (N=611)
Patients with any TEAE	592 (97.9%)	606 (99.2%)
Patients with any grade 3-4 TEAE	378 (62.5%)	510 (83.5%)
Patients with any grade 3-4 related TEAE	284 (46.9%)	451 (73.8%)
Patients with any serious TEAE	198 (32.7%)	294 (48.1%)
Patients with any serious related TEAE	93 (15.4%)	194 (31.8%)
Patients with any TEAE with a fatal outcome[a]	29 (4.8%)	37 (6.1%)
Any patient who permanently discontinued due to TEAE	73 (12.1%)	164 (26.8%)

n(%) = number and percentage of patients with at least one TEAE

TEAE: Treatment-Emergent Adverse Event

(a) based on AE reported start date

BENEFIT RISK SUMMARY CONT:

Summary 6.1 Weights and values

Benefits	Relative Importance (weighting)	Valuing the options: Investigated product	Comparator	Placebo	Comment on strength and uncertainty of benefit
Overall survival	Not available in Overall conclusion and risk/benefit assessment	13.5 months		12.1 months	The sponsor notes17 that "the objective of subgroup analyses in the study was to assess the consistency of the treatment effect across subgroups" and also that "the study was not powered to demonstrate a statistically significant treatment effect in a particular subgroup". In unadjusted sub-group analysis, there is an indication of an inconsistent treatment effect across subgroups defined by ECOG status, with the ECOG 0 subgroup results favouring aflibercept, the ECOG 1 subgroup results similar across arms, and the ECOG 2 subgroup results favouring placebo (there, median OS was 4.4 months in the placebo arm and 2.8 months in the aflibercept arm). A test for heterogeneity of treatment effects was negative18; this was taken to support "a consistent effect of treatment across subgroups" (however given the actual results, this negative test for heterogeneity does not seem to be strong evidence for a consistent treatment effect). It is accepted that these are exploratory results and that the ECOG 2 stratum was small (27/1226); also, this pattern was not repeated for PFS data. The sponsor discusses ECOG 0-1 results but not ECOG 2 results in the Clinical Trials section of the draft PI, but in light of the above this is probably reasonable.
Progression-free survival	Not available in Overall conclusion and risk/benefit assessment	6.9 months		4.7 months	Not available in Overall conclusion and risk/benefit assessment
Objective response rate	Not available in Overall conclusion and risk/benefit assessment	19.8%		11.1%	Not available in Overall conclusion and risk/benefit assessment

Please describe methodology used for assessing relative importance: eg Ranking or point allocation and also what is has been used in relation to valuing the options e.g. % change, Number of patients, etc

Not available in Overall conclusion and risk/benefit assessment

BENEFIT RISK SUMMARY CONT:

Risks	Relative Importance (weighting)	Valuing the options Investigated product	Comparator	Placebo	Comment on strength and uncertainty of each risk	Was the value or weight of this risk altered or mitigated by the ability to control the use of the medicine once on the market?
Deaths	Not available in Overall conclusion and risk/benefit assessment	66.0%		75.7%	Not available in Overall conclusion and risk/benefit assessment	Not available in Overall conclusion and risk/benefit assessment
Hypertension	Not available in Overall conclusion and risk/benefit assessment	41.2%		10.7%	Study PDY6655 confirmed that aflibercept increases blood pressure.	Not available in Overall conclusion and risk/benefit assessment
QT prolongation	Not available in Overall conclusion and risk/benefit assessment	NA		NA	Not available in Overall conclusion and risk/benefit assessment	Not available in Overall conclusion and risk/benefit assessment
Haemorrhage	Not available in Overall conclusion and risk/benefit assessment	37.8%		19.0%	Not available in Overall conclusion and risk/benefit assessment	Not available in Overall conclusion and risk/benefit assessment
Proteinuria	Not available in Overall conclusion and risk/benefit assessment	62.7%		40.7%	Despite the frequency of proteinuria, there was no strong signal of associated renal impairment (for example, renal failure events were reported in 2.9% of aflibercept and 2.1% of placebo patients).	Not available in Overall conclusion and risk/benefit assessment
Diarrhea and stomatitis	Not available in Overall conclusion and risk/benefit assessment	19.3% (diarrhea), 50.1% (stomatitis)		7.8% (diarrhea), 32.9% (stomatitis)	Not available in Overall conclusion and risk/benefit assessment	Not available in Overall conclusion and risk/benefit assessment
Fistulae and gastrointestinal perforation	Not available in Overall conclusion and risk/benefit assessment	9 (fistula), 0.8% (perforation)		3 (fistula), 0.3% (perforation)	Not available in Overall conclusion and risk/benefit assessment	Not available in Overall conclusion and risk/benefit assessment
Thromboembolism	Not available in Overall conclusion and risk/benefit assessment	2.6%		1.5%	Not available in Overall conclusion and risk/benefit assessment	Not available in Overall conclusion and risk/benefit assessment
Blood cell counts	Not available in Overall conclusion and risk/benefit assessment	Slight increase in neutropenia, thrombocytopenia. Slight decrease in anemia			Not available in Overall conclusion and risk/benefit assessment	Not available in Overall conclusion and risk/benefit assessment
Hepatic and renal toxicity	Not available in Overall conclusion and risk/benefit assessment	2.9% (renal failure)		2.1% (renal failure)	Not available in Overall conclusion and risk/benefit assessment	Not available in Overall conclusion and risk/benefit assessment
Dysphonia	Not available in Overall conclusion and risk/benefit assessment	NA		NA	Anti-VEGF drugs have been described based on study of 5 patients as causing laryngeal mucosal pathology akin to that seen in the nasal mucosa. Dysphonia is described as commonly reported in the post-market setting for bevacizumab.	Not available in Overall conclusion and risk/benefit assessment
Immunogenicity	Not available in Overall conclusion and risk/benefit assessment	1.3%		0.2%	Not available in Overall conclusion and risk/benefit assessment	Not available in Overall conclusion and risk/benefit assessment

Please describe methodology used for assessing relative importance: eg Ranking or point allocation and also what is has been used in relation to valuing the options e.g. % change, Number of patients, etc

Not available in Overall conclusion and risk/benefit assessment

BENEFIT RISK SUMMARY CONT:

Summary 7.1 Conclusion

**Summary 7.1.1** If the benefit-risk balance is assessed to be negative, describe the harm (e.g. in terms of lack of efficacy, toxicity) that the drug may cause if used in the proposed indication
Not applicable, as Delegate's opinion was an overall positive benefit-risk balance. Rationale of clinical evaluator's negative opinion found only in main clinical report and not found in this section of overall conclusion.
**Summary 7.1.2** Describe how the benefit-risk balance is expected to evolve over time (e.g. when late side effects emerge or long-term efficacy decreases)
Sponsor's comments: The Kaplan-Meier survival curves continue to separate past the median time point indicating that the magnitude of the aflibercept treatment effect is increasing over time.
**Summary 7.1.3** Describe outstanding issues, and other significant information eg, submission of additional reports by the company to address those issues, hearings and advisory group recommendations, information from other jurisdictions (eg advisory committees, scientific experts, patients, consumers, consumer advocates and other stakeholders)
The Delegate sought general advice on this application from the Advisory Committee on Prescription Medicines (ACPM), and in particular requested the committee consider the value of either adding an explanatory sentence to the indication.
**Summary 7.1.4** Make reference to the evaluation of the pharmacovigilance plan and risk minimization plan if any. Describe any communication or particularly significant information to the medical profession, patients or the public that is required. Describe restrictions to product availability or usage
The Delegate has indicated that the proposed condition of registration include:<br>· EU RMP v1.0 dated 18 October 2011 and the ASA version 1.0 dated March 2012<br>An updated ASA version 1.1 has been submitted to the TGA and a further update is included with this response to reflect the latest EU-RMP and the EU SmPC. The sponsor suggested the conditions of registration should therefore refer to:<br>· EU RMP v1.3 dated 15 November 2012 and ASA version 1.2 dated January 2013<br>In relation to osteonecrosis mentioned in the Delegate's overview, an assurance is provided that if any post-marketing signals of disproportionately high reporting is received (especially if cases are not associated with bisphosphate use), osteonecrosis will be reconsidered early and if necessary the PI will be amended accordingly.
**Summary 7.1.5** Describe the need for further studies (e.g. the need for studies to improve the benefit-risk balance with further optimization studies, the need for intensive additional follow up measures or specific obligations, and the need for further development including any paediatric development plans.
Nil

Summary 7.1.6 Please provide any other information considered by the agency relevant to the benefit risk decision that is not covered elsewhere in the proforma.

Delegate considerations
Efficacy
The clinical evaluator states that "results are considered to be clinically insignificant, based on the survival parameters used to calculate the sample size". The Delegate considers that OS results are statistically and clinically significant, in the context of available treatments. Sample size criteria are used to design an adequately powered study. In the Delegate's view, the minimum clinically significant difference in OS may differ from the parameters used to calculate sample size.
There is only one randomised, controlled study to support efficacy. The TGA's guidance on applications with one pivotal study states that "the minimum requirement is generally one controlled study with statistically compelling and clinically relevant results". Section III.2 of this guideline notes characteristics of acceptable single pivotal studies. The Delegate's view is that VELOUR can be considered an acceptable single pivotal study.

The Delegate proposed to approve the application with the indication proposed by the sponsor.

The Delegate sought general advice on this application from the ACPM, and in particular requested the committee consider the value of either adding an explanatory sentence to the indication (for example, "For second line treatment for metastatic colorectal cancer, there are clinical trial data to support use of aflibercept in combination with FOLFIRI, but none to support use in combination with other irinotecan-fluoropyrimidine-based regimens") or referring directly to FOLFIRI (compare with the US indication).

Patients with MCRC who have progressed following an oxaliplatin-based treatment regimen have few therapeutic options. The sponsor endorses the Delegate's recommendation to approve Zaltrap for use in this indication which will address an unmet clinical need. It should be noted that aflibercept was approved in combination with FOLFIRI by the FDA on August, 3, 2012 and the use of aflibercept in combination with FOLFIRI in this patient setting has also been included in the National Comprehensive Cancer Network (NCCN) guidelines which are widely recognised and referenced in Australian clinical practice. In the EU, a positive CHMP Opinion was issued in November 2012 to recommend approval.

Summary 7.1.7 Please provide a clear conclusion on the benefit-risk being positive or not for the proposed indication.

The Advisory Committee on Prescription Medicines (ACPM), having considered the evaluations and the Delegate's overview, as well as the sponsor's response to these documents, advised the following:
The ACPM, taking into account the submitted evidence of efficacy, safety and quality considered these products to have an overall positive benefit–risk profile.

Summary 7.1.8 Please provide the indication recommended following the outcome of the benefit-risk balance.

Based on a review of quality, safety and efficacy, TGA approved the registration of Zaltrap/Alfitiv/Lidaveg concentrated injection vial containing aflibicept rch 4 mg/100 mL and 8 mg/200 mL for intravenous infusion, indicated for:
Zaltrap/Aflitiv/Lidaveg in combination with irinotecan-fluoropyrimidine-based chemotherapy is indicated in adults with metastatic colorectal cancer previously treated with an oxaliplatin-containing regimen.

Reviewers Name:	Not available in Overall conclusion and risk/benefit assessment
Signature:	Not available in Overall conclusion and risk/benefit assessment
Date:	Not available

Manager sign-off or Peer review

Reviewers Name:	Not available in Overall conclusion and risk/benefit assessment
Signature:	Not available in Overall conclusion and risk/benefit assessment
Date:	Not available
